# Pregnancy in a noncommunicating rudimentary horn of a unicornuate uterus: a case report

**DOI:** 10.1186/1757-1626-2-6624

**Published:** 2009-04-27

**Authors:** Patrick I Okonta, Harrison Abedi, Caroline Ajuyah, Lawrence Omo-Aghoja

**Affiliations:** 1 Department of Obstetrics and Gynaecology, Faculty of Medicine, College of Health SciencesDelta State University, Abraka, Delta StateNigeria; 2 Department of Medicine, Central HospitalWarri, Delta StateNigeria

## Abstract

Pregnancy in the rudimentary horn is rare and carries grave consequences for the mother and fetus. A case report is presented of a 26 year old single gravida 3 para 0^+2^ lady with rupture of a rudimentary horn pregnancy at a gestational age of 20 weeks. Laparotomy was done and the rudimentary horn excised. Post-operative recovery was uneventful. The need for a high index of suspicion and the role of ultrasonography in the accurate diagnosis is highlighted.

## Introduction

Pregnancy in a rudimentary horn of a unicornuate uterus is rare [1]. An incidence of 1 in 76,000 - 150,000 pregnancies is reported in the literature [2,3]. We present a case report of rupture of a 21 weeks pregnancy in the non-communicating rudimentary horn of a unicornuate uterus in a Nigerian woman

## Case presentation

The patient is a 26-year-old single gravida 3 para 0^+2^ Nigerian trader. She was admitted into the gynaecology ward through the accident and emergency unit of our hospital. She was pregnant with an estimated gestational age of 20 weeks.

Her presenting complaints were abdominal pain of 4 days duration and vomiting of a day's duration. Abdominal pain started after she had gone for her regular abdominal massage in a traditional birth attendant's home. It was moderately severe and relieved mildly by analgesics. She vomited twice before presentation. Vomiting was non-projectile and not provoked by oral intake. Bowel opening was normal. There was associated dizziness and she felt faint. However, there was no bleeding per vagina.

She had attempted terminating this pregnancy thrice at a gestational age of 6, 8 and 11 weeks respectively by dilatation and curettage without success in a private medical clinic. She had not done any previous ultrasound scan.

At presentation, she looked pale and had a tinge of jaundiced. Her height and weight was not recorded. Her pulse was 120 beats per minute and of low volume. The blood pressure was 100/50 mmHg. The abdomen was enlarged with generalised tenderness. There was a suprapubic mass corresponding to a 22 weeks size uterus. There was no bleeding per vagina. A diagnosis of an acute abdomen with heamoperitoneum from a possible extra uterine pregnancy was made.

The packed cell volume was 19% and immediate blood transfusion was commenced, urgent ultrasound scan was requested and laparotomy planned.

Abdominal ultrasound scan done was reported as follows:

“A single viable intrauterine fetus with normal cardiac activity is noted. The BPD is 48mm corresponding to a gestational age of 19 weeks and 6 days. There is also a contracted liver with increased echogenicity and surrounded by ascitic fluid.

Conclusion:

1. A single viable intrauterine pregnancy of about 20 weeks.
2. Chronic liver disease? Cirrhosis.”

Laparotomy was withheld and the medical team invited to evaluate the patient. Liver enzymes were elevated and total protein was reduced. A diagnosis of Septicaemia to rule out Malaria and Hepatitis was made by the medical team.

She was given parenteral antibiotics, anti-malarial, fluids and analgesics. There was a marginal transient improvement in her condition. A subsequent repeat abdominal ultrasound scan was requested which showed:

“Marked ascites with a contracted liver. There is a non-viable intra-abdominal fetus. There is a bulky non-gravid anteverted uterus.

Conclusion:

1. Non-viable intra-abdominal pregnancy of about 20 weeks.
2. Cirrhosis of the liver.”

**Figure 1.**
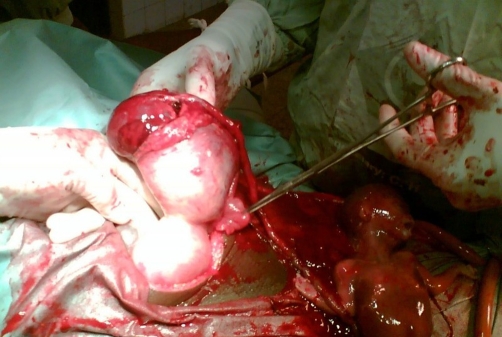
Intra-operative photograph showing the anterior view of the uterus with the rudimentary horn attached to its left superior border. The rudimentary horn has a 5 cm rupture with the placenta partially protruding through it. The fetus is already extruded from the horn with the umbilical cord still attached to it on one end and the placenta in the horn.

She subsequently had Laparotomy. Findings at Laparotomy were a haemoperitoneum of about 1.5 litres, and a dead fetus floating in the peritoneal cavity. The fetus weighed 350 grams. There was a left rudimentary horn of the uterus with a 5 cm rupture on the superior margin. The placenta was still within the uterine horn. The cavity of the horn did not communicate with the uterine cavity. The left fallopian tube was of normal length and attached to the rudimentary horn. Left ovary was normal and attached by its ligament to the rudimentary horn. The uterus was of normal size with the right fallopian tube and right ovary attached to it (Figure 1).

Excision of the rudimentary horn and the left fallopian tube was done. The left ovary was conserved. She was transfused with 2 pints of blood intraoperatively. Her post operative recovery was normal. She was discharged on the 7^th^ post operative day and given a 6 weeks follow-up appointment when she will do an intravenous urogram. She however did not turn up for her appointment.

## Discussion

Rudimentary horn with a unicornuate uterus results from failure of complete development of one of the mullerian ducts and incomplete fusion with the contralateral side. In 83% of cases the rudimentary horn is non-communicating [4].

Pregnancy in a non communicating rudimentary horn occurs through transperitoneal migration of sperm or fertilized ovum [5]. It is associated with a high rate of spontaneous abortion, preterm labour, intrauterine growth retardation, intraperitoneal haemorrhage and uterine rupture [6]. Diagnosis prior to rupture is unusual, but could be made with ultrasonography and MRI. Tsafrir *et al* outlined a set of criteria for diagnosing pregnancy in the rudimentary horn [7]. They are: (1) A pseudo pattern of asymmetrical bicornuate uterus; (2) Absent visual continuity tissue surrounding the gestation sac and the uterine cervix: (3) Presence of myometrial tissue surrounding the gestation sac. None-the-less most cases remain undiagnosed until it ruptures and presents as an emergency.

The patient presented at a G.A. of 21 weeks with clinical features suggestive of a ruptured extrauterine pregnancy. However, the initial ultrasound scan indicated that the pregnancy was viable and intrauterine and that she had a cirrhotic liver. This caused some diagnostic dilemma and the immediate laparotomy planned was withheld. Probably the diagnosis was initially missed on ultrasound due to a poor index of suspicion. Furthermore the fetus was still viable then and had not been extruded from the rudimentary horn. Her clinical condition did not improve remarkable and the diagnosis of an acute abdomen from possible ruptured extrauterine pregnancy was still entertained despite the initial ultrasound report. Fortunately, this was confirmed by a repeat scan and she had laparotomy subsequently. The importance of correct and accurate ancillary investigative reports to collaborate clinical diagnosis cannot be over-emphasized.

The usual outcome of rudimentary horn pregnancy is rupture in second trimester in 90% of cases with fetal demise [8], however cases of pregnancy progressing to the third trimester and resulting in a live birth after caesarean section has been documented [6].

Interesting to note is the fact that the patient had tried unsuccessfully thrice to terminate her pregnancy. Continued pregnancy after prior attempts at termination by dilatation and curettage is often associated with a missed diagnosis of an extrauterine pregnancy.

It is recommended by most that immediate surgery be performed whenever a diagnosis of pregnancy in a rudimentary horn is made even if unruptured [9]. However, conservative management until viability is achieved has been advocated in very select cases with larger myometrial mass, if emergency surgery can be performed anytime and the patient is well-informed [10].

Pregnancy in a rudimentary horn carries grave risk to the mother. There is need for increased awareness of this rare condition and to have a high index of suspicion especially in developing countries where the possibility of early detection before rupture is unlikely.

## Abbreviations

BPD: Biparietal diameter
cm: Centimetres
GA: Gestational age
mm: Millimetres
mmHg: Millimetres of mercury
